# Whole-Brain Source-Reconstructed MEG-Data Reveal Reduced Long-Range Synchronization in Chronic Schizophrenia

**DOI:** 10.1523/ENEURO.0338-17.2017

**Published:** 2017-10-17

**Authors:** Jonni Hirvonen, Michael Wibral, J. Matias Palva, Wolf Singer, Peter Uhlhaas, Satu Palva

**Affiliations:** 1 Helsinki Institute of Life Sciences, Neuroscience Center, 00014 University of Helsinki, Finland; 2 MEG-Unit, Goethe-University, 60323 Frankfurt, Germany; 3 Department of Neurophysiology, Max Planck Institute for Brain Research, 60438 Frankfurt am Main, Germany; 4 Ernst Strüngmann Institute for Neuroscience (ESI) in Cooperation with Max Planck Society, 60528 Frankfurt am Main, Germany; 5 Frankfurt Institute for Advanced Studies (FIAS), 60438 Frankfurt am Main, Germany; 6 Institute of Neuroscience and Psychology, University of Glasgow, G12 8QB Glasgow, United Kingdom

**Keywords:** MEG, oscillation, perception, schizophrenia, synchronization

## Abstract

Current theories of schizophrenia (ScZ) posit that the symptoms and cognitive dysfunctions arise from a dysconnection syndrome. However, studies that have examined this hypothesis with physiological data at realistic time scales are so far scarce. The current study employed a state-of-the-art approach using Magnetoencephalography (MEG) to test alterations in large-scale phase synchronization in a sample of *n* = 16 chronic ScZ patients, 10 males and *n* = 19 healthy participants, 10 males, during a perceptual closure task. We identified large-scale networks from source reconstructed MEG data using data-driven analyses of neuronal synchronization. Oscillation amplitudes and interareal phase-synchronization in the 3–120 Hz frequency range were estimated for 400 cortical parcels and correlated with clinical symptoms and neuropsychological scores. ScZ patients were characterized by a reduction in γ-band (30–120 Hz) oscillation amplitudes that was accompanied by a pronounced deficit in large-scale synchronization at γ-band frequencies. Synchronization was reduced within visual regions as well as between visual and frontal cortex and the reduction of synchronization correlated with elevated clinical disorganization. Accordingly, these data highlight that ScZ is associated with a profound disruption of transient synchronization, providing critical support for the notion that core aspect of the pathophysiology arises from an impairment in coordination of distributed neural activity.

## Significance Statement

Despite over 100 years of research, the pathophysiology of schizophrenia (ScZ) has remained elusive. Synchronization of neuronal activity across brain regions, a form of functional connectivity, is crucial for normal brain functioning. We tested the hypothesis that disruption of connectivity and synchronization could lead to the cognitive deficits in ScZ by recording magnetoencephalography (MEG) during a visual perceptual closure task. Long-range high-frequency synchronization in β- and γ-bands was reduced in chronic schizophrenia patients compared to healthy controls. This reduction of neuronal synchronization showed close correlations with the severity of clinical signs of cognitive disorganization. Reduced synchronization may thus constitute a core pathophysiological mechanism in ScZ.

## Introduction

Despite over 100 years of research, the pathophysiology of schizophrenia (ScZ) remains elusive. Previous theoretical and empirical frameworks explain the disorder as circumscribed alterations in neural circuits (Weinberger et al., 1988). An alternative hypothesis suggests that core aspects of symptoms and associated cognitive disturbances arise from a deficit in the functional integration of distributed brain networks leading to a dysconnection syndrome (Stephan et al., 2009). This hypothesis is supported by extensive evidence from normal brain functioning, suggesting that functional interactions between distributed neuronal ensembles are critical for the generation of coherent action and cognition (Singer, 1999; Varela et al., 2001). One mechanism to achieve such interactions is the synchronization of rhythmic activity that could promote effective coordination of neuronal processing (Gregoriou et al., 2009; Singer, 2009; Fries, 2015).

Given the crucial role of synchronization for effective brain functioning, one possibility is that a disruption in this process leads to behavioral and cognitive deficits observed in ScZ (Uhlhaas and Singer, 2015). Evidence from electro- and magnetoencephalography (EEG/MEG) has provided support for the possibility that both the amplitude of high-frequency oscillations and their long-range synchronization in relationship to perceptual processing are impaired (Uhlhaas, 2015). Several previous studies on perceptual integration have focused on the possibility that local neuronal synchronization, reflected by the amplitude/power of oscillatory activity, may be reduced at β (14 − 30 Hz) and γ-band frequencies along different stages of the visual hierarchy (Spencer et al., 2003; Uhlhaas et al., 2006; Spencer et al., 2008; Grützner et al., 2013; Grent-'t-Jong et al., 2016). Moreover, preliminary data also suggest abnormalities in long-range synchronization during perceptual integration (Spencer et al., 2003; Uhlhaas et al., 2006). These lines of research are consistent with evidence that synchronization of high-frequency oscillations may be associated with construction of coherent object representations during normal brain functioning (Keil et al., 1999; Rodriguez et al., 1999; Grützner et al., 2010), and that disturbance in γ-band synchronization may be at the root of the pervasive perceptual deficits in ScZ (Uhlhaas and Mishara, 2007; Uhlhaas and Singer, 2015).

A key limitation of most prior studies in ScZ is that estimates of neuronal synchronization were derived from scalp-EEG data. Because of volume conduction, individual EEG electrodes pick up signals from multiple neuronal and non-neuronal sources, such as muscles, which can give rise to artifacts and spurious correlations that yield false positives and mask true neuronal interactions (Nolte et al., 2004; Schoffelen and Gross, 2009; Brookes et al., 2012; Palva and Palva, 2012). These effects can be alleviated by using MEG data together with source reconstruction and synchronization metrics that are less sensitive to signal contamination and volume conduction (Schoffelen and Gross, 2009; Palva and Palva, 2012). Because of these methodological limitations, it is also currently unclear at which spatial scale and between which brain regions the synchronization deficits occur in ScZ, i.e., whether the putative anomalies are restricted to local cortical areas or involve also large-scale interareal neuronal interactions.

To overcome these limitations, we analyzed large-scale synchronization in a group of chronic ScZ patients using MEG and applied a data-driven, whole-brain analysis of MEG activity (Palva and Palva, 2010) obtained during a perceptual closure task. The data presented here were previously analyzed for MEG-sensor-level changes in β/γ-band power (Grützner et al., 2013). MEG has an advantage over EEG in having a greater signal-to-noise ratio for high-frequency oscillations (Muthukumaraswamy and Singh, 2013) and better spatial resolution for localizing the underlying generators (Dale and Sereno, 1993; Sharon et al., 2007). Large-scale networks of neuronal synchronization were estimated among all parcels (brain areas) using source-modeled MEG. Parcellated data were then tested for modulations in within-parcel spectral power and interparcel phase-synchronization in the 3–120 Hz frequency range that was correlated with clinical symptoms and neuropsychological scores. We found that the ScZ patients were characterized by a reduction in γ-band amplitude (30–40 and 60–120 Hz) that was accompanied by a pronounced deficit in large-scale synchronization at β/γ-band frequencies. These abnormalities showed close correlations with the severity of clinical signs of cognitive disorganization.

## Materials and Methods

**Figure 1.**
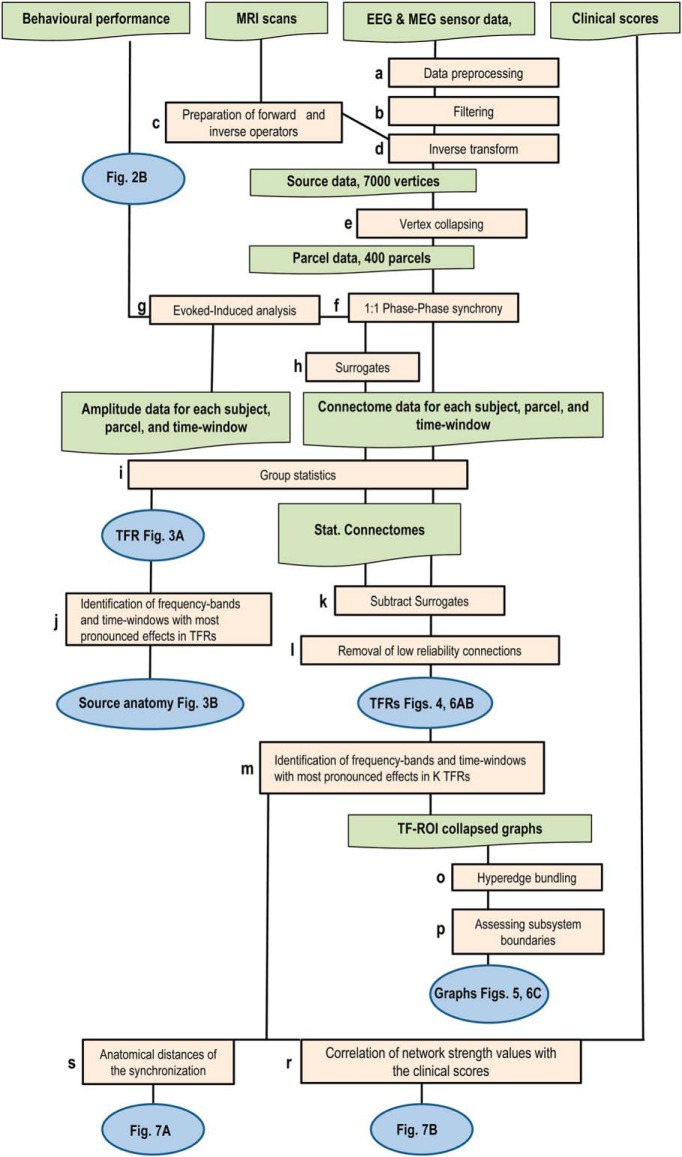
A schematic overview of the analysis pipeline showing the different analysis steps and outputs of the results (for a-, see Materials and Methods).

An overview of the MEG-analysis pipeline is given in Figure 1. All data analyses, where not indicated otherwise, were performed on a LabVIEW-based (National Instruments) neuroinformatics platform that is available on request.

### Subjects and recordings

We recruited 18 medicated ScZ patients who met the DSM-IV criteria for ScZ from the Department of Psychiatry, Johann Wolfgang Goethe University, Frankfurt; Germany. Two patients had to be excluded because of missing channel data, leaving 16 patients (10 males, three left handed, mean age 37.06 ± 9.8). We also recruited 19 age- and gender-matched healthy control subjects (13 males, four left handed, mean age 32.42 ± 10.6) from the local community. All patients were on atypical neuroleptics at the time of testing. DSM-IV diagnosis for ScZ was confirmed by a trained psychologist with the SCID-interview for DSM-IV-R (First 1995). All ScZ patients were on stable neuroleptic medication. Exclusion criteria were for both ScZ patients and controls: (1) a neurologic disorder; (2) alcohol, nicotine, or substance dependence within the last month; or (3) structural abnormalities in the T1 MR image. After having received a complete description of the study, each participant provided written informed consent. The study was approved by the ethical committee of the Goethe University Frankfurt.

Current psychopathology was assessed with the Positive and Negative Syndrome Scale (PANSS; Kay et al., 1987) and symptoms were grouped into five factors according to the model of (Lindenmayer et al., 1995), including the factors “positive,” “negative,” “depression,” “excitement,” and “cognitive.” Cognitive function in patients and controls was measured with the Brief Assessment of Cognition in Schizophrenia (BACS; Keefe et al., 2004; Table 1).

**Table 1.** Means, SDs, and mean differences for demographic, neurocognitive, and clinical characteristics of controls and ScZ patients

	Healthy controls (*N* = 19)			Chronic patients (*N* = 16)			Statistics	
Basics	Mean		SD	Mean		SD	χ^2^/*t* value	*p* value
Gender (m/f)		13/6			10/6		χ^2^_1_ = 1.01	0.71
Age	32.42		10.61	37.06		9.47	*t*_(33)_ = −1.37	0.18
Education	15.5		3.17	13.94		3.30	*t*_(31)_ = 1.16	0.25
Handedness	76		42.06	77		42.13	*t*_(29)_ = −0.03	0.97
BACS								
Verbal Memory	51.61		7.06	37.87		14.40	*t*_(30)_ = 3.43	0.0018
Digit	24.56		3.99	19.53		4.41	*t*_(30)_ = 3.22	0.0031
Motor	89.65		9.03	74.87		11.09	*t*_(29)_ = 3.99	0.0004
Fluency	58.83		13.7	42.13		9.21	*t*_(30)_ = *2*.96	0.0060
Symbol cod.	55.67		14.84	46.87		15.72	*t*_(30)_ = 1.57	0.1269
ToL	19.83		2.2	18.07		2.71	*t*_(30)_ = 2.07	0.0467
PANSS								
Negative	−		–	16.6		4.76	–	–
Excitement	–		–	6.07		1.83	–	–
Positive	–		–	9.4		3.98	–	–
Cognition	–		–	9.6		3.14	–	–
Depression	–		–	12.67		3.62	–	–
Disorganization	–		–	5.53		2.00	–	–
Total Score	–		–	238.84		43.09	–	–

MEG data were recorded continuously using a 275-channel whole-head system (Omega 2005, VSM MedTech) at a rate of 600 Hz in a synthetic third order axial gradiometer configuration (Data Acquisition Software version 5.4.0, VSM MedTech). The data were filtered with 4th order butterworth filters with 0.5 Hz high-pass and 150 Hz low-pass. Behavioral responses were recorded using a fiber-optic response pad (Lumitouch, Photon Control) on the stimulus PC and fed through to the MEG acquisition system as an additional channel. Before and after each run, the subject’s head position relative to the gradiometer array was measured using coils placed at the subject’s nasion, and 1 cm anterior to the tragus of the left and right ear. Runs with total head displacement exceeding 5 mm were discarded.

### Anatomic (MRI) data acquisition

A high-resolution anatomic MRI scan was acquired for each participant using a 3D magnetization-prepared rapid-acquisition gradient echo sequence (160 slices; voxel size: 1 × 1 × 1 mm; FOV: 256 mm; TR: 2300 ms; TE: 3.93 ms). During the structural scan, vitamin E pills were applied to the nasion and 1 cm anterior to the tragus of the right and left ear to allow for coregistration of the MEG and MRI data. Scanning was performed with a 3 Tesla Siemens Trio scanner.

### Experimental protocol

**Figure 2.**
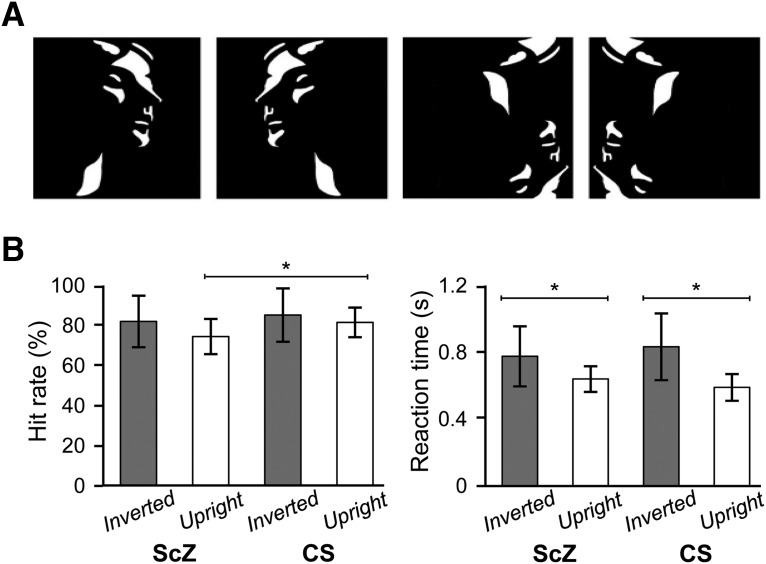
Stimuli and behavioral performance in ScZ patients and controls (CS). ***A***, Upright (top) and inverted (bottom) Mooney face stimuli used in the task. ***B***, HRs and RTs for both groups for the upright and inverted stimuli. Lines indicate significant difference between the groups (*p < 0.01, one-way ANOVA, Bonferroni corrected at α = 0.05/4).

We used data from our previous study in which experimental procedures have been described (Grützner et al., 2013). In brief, we presented a set of 160 Mooney faces (Mooney and Ferguson, 1951), consisting of the 40 original Mooney stimuli presented in the upright orientation, mirrored at the vertical axis and in corresponding versions mirrored at the horizontal axis. Participants were presented with a random sequence of upright and inverted-scrambled stimuli which were shown for 200 ms (Fig. 2*A*). The interstimulus interval (ISI) ranged between 3500 and 4500 ms. Participants were required to indicate whether they detected a face or not via button press after each stimulus. They were instructed to respond as quickly as possible and to fixate a central fixation cross during the ISI. All participants completed four experimental runs, each of which was composed of 60 upright and 30 inverted scrambled stimuli. The stimuli were displayed in the center of a translucent screen at a viewing distance of 53 cm and subtended 19° of visual angle.

### Behavioral performance

Hit rate (HR) was estimated as the proportion of correct responses from all responses to upright and inverted-scrambled Mooney-face stimuli. The correct responses were “no face” for inverted-scrambled Mooney faces and “face” for upright stimuli. The latency at which either face or no face button was pressed was defined as the reaction time (RT).

### MEG data preprocessing, filtering, source analysis, and surface parcellation

Extracranial noise from the raw MEG recordings was removed with the temporal signal space separation method (tSSS; Taulu et al., 2005) and independent component analysis (ICA; Bell and Sejnowski, 1995) was used to identify and exclude components associated with eyes movements/blinks and cardiac artifacts (Fig. 1*A*). The preprocessed MEG time series data from each separate channel was then narrow-band filtered into 31 frequency bands, *f_min_* = 3 Hz *… f_max_* = 120 Hz by convolving the sampled MEG signals with a family of Morlet wavelets with *m* = 5 (Fig. 1*B*). Finite impulse-response filter was used for broad-band filtering from 0.1–45 Hz (pass-band from 1–40 Hz) and Hilbert-transformation to obtain the signal phase time series (Palva et al., 2013) for the evoked responses (ERs; Fig. 4*A*).

We used FreeSurfer software (http://surfer.nmr.mgh.harvard.edu/) for automatic volumetric segmentation of the MRI data, surface reconstruction, flattening, cortical parcellation, and labeling with the Freesurfer/Destrieux atlas (Dale et al., 2000; Lin et al., 2006; Destrieux et al., 2010). MNE software (http://www.nmr.mgh.harvard.edu/martinos/userInfo/data/sofMNE.php) was used to create three-layer boundary element models (BEMs), cortically constrained source models, MEG-MRI colocalization and for the preparation of the forward model and inverse operators (Hämäläinen and Ilmoniemi, 1994; Gramfort et al., 2014). The source models had dipole orientations fixed to the pial surface normals and a 7-mm source-to-source separation throughout the cortex yielding 6000–8000 source vertices. To reconstruct ongoing cortical dynamics, we used minimum-norm estimate (MNE) inverse operators in the form of dynamic statistical parametric maps (dSPM; Dale et al., 2000) by computing noise-covariance matrices from the baseline (0.5-0.1 s before stimulus onset) and using 0.05 as the regularization constant (Fig. 1*C*,*D*). The source time series were collapsed to time series of 400 cortical parcels with a fidelity optimized collapse operator (Korhonen et al., 2014) from a precursor atlas of 148 parcels (Destrieux et al., 2010) by iteratively splitting the largest parcels of the Destrieux atlas along their most elongated axis using the same parcel-wise splits for all subjects (Fig. 1*E*).

### Analysis of oscillation amplitudes, ERs, and interareal phase synchrony

The collapsed parcel-wise narrow-band inverse estimates, X_F,P,_*_r_*(*t*,*f*) of single trials *r*, *r* = 1… *n_s_*, were used for cortex-wide mapping of ERs (Fig. 1*G*), induced amplitude modulations, and interareal phase synchronization (Fig. 1*F*). The averaged event-related amplitude envelopes were estimated separately for each trial type (upright correct, inverted correct, upright incorrect, inverted incorrect), cortical parcel, and wavelet frequency (Tallon-Baudry et al., 1996; Palva et al., 2005). The filtered ERs were obtained by the averaging the real parts of the complex filtered parcel time series.

Phase synchrony was estimated in 100-ms time windows with 50-ms overlap separately for the same trial types as above for all frequencies and for all parcel pairs using the imaginary part of complex form of PLV (iPLV) to maximally attenuate artificial interactions caused by linear signal mixing (Vinck et al., 2011). Since iPLV is sensitive to the number of trials, the trial number was balanced separately for each subject and for each contrast before the analyses by iteratively removing trials from the condition with more trials to match the condition with fewer trials s (Fig 1*F*). On average, 184 ± 38 (mean ± SD) and 104 ± 25 trials were obtained for the ScZ patients with correctly perceived upright and inverted-scrambled stimuli, respectively. The control group had 234 ± 57 and 126 ± 38 trials in upright and inverted-scrambled conditions, respectively.

To distinguish between synchronization caused by stimulus onset (evoked synchrony) and the dynamically generated synchronisation/phase locking (stimulus induced synchrony), we created trial-shuffled surrogate data for shift–predictor like estimation of stimulus-driven synchronization. However, in addition to eliminating nonstimulus-locked synchronization (Lachaux et al., 1999), trial shuffling alone also eliminates the signal-mixing caused artificial couplings. to reconstruct the effects of signal mixing at MEG acquisition and the residual signal leakage after inverse modeling, we applied a novel forward-inverse-modeling based approach. This method eliminates confounds caused by the spatial spread of signals inherent in MEG/EEG recordings and reconstructs the signals preserving local source topography, amplitude dynamics and auto-correlation structures (Palva and Palva, 2012). This new approach permits more accurate identification of “true” induced interareal interactions in the presence of signal mixing than the conventional trial shuffling procedure (Lachaux et al., 1999) that eliminates also the contributions of volume conduction and other signal mixing. We first used shuffled trials of source-modeled single-trial data in the 400-parcel time series for forward modeling, so that each source vertex of a parcel was simulated with this time series, and then source reconstructed these sensor-level surrogate data with procedures identical to those used for real data (Fig. 1*H*). Phase correlation analyses were then applied to these surrogate source data in the same way as to the real data for 10 independent realizations of the surrogate data. The means of surrogate data were subtracted directly from the corresponding real data in Figures 4-7 (Fig. 1*K*).

### Statistical analyses and visualization (of the most significant effects)

We used statistical testing across all brain regions, frequency bands, and time windows to reveal the task-related amplitude and synchrony modulation. Before performing statistical group analyses for amplitude, individual data were baseline corrected parcel-by-parcel by subtracting from all samples the mean amplitude of a baseline period from 0.5-0.1 s before the stimulus onset. For the synchrony analyses, the iPLV values of the baseline time window at 0.225 − 0.125 s before stimulus onset were used for baseline correction. Significant differences between the responses to upright/inverted stimuli and the baseline period as well as between the upright and inverted stimuli were estimated with the Wilcoxon signed-rank test (*p* < 0.05). For the between-group comparisons, the Welch *t* test was used separately for the upright and inverted conditions (Fig. 1*i*). In all analyses, only trials with correct responses were used. To reduce the false discovery rate (FDR) for each contrast, we pooled significant observations across all samples, frequency bands and cortical parcels and then discarded as many least-significant observations as were predicted to be false discoveries by the α-level used in the corresponding test (Rouhinen et al., 2013; Siebenhühner et al., 2016).

To obtain a data-driven overview of all significant observations, we plotted for the amplitude data the fractions of parcels out of all 400 parcels exhibiting a statistically significant positive or negative effect (*P^+^_P_* or *P* ^−^*_P_*) for each time frequency (TF) element in the peri-event TF plane. Likewise, to assess the extent of large-scale synchronization in each frequency band and time window, we defined connection density *K* to be the fraction of significant edges of all possible edges (*K = k/(N−1)N,* where *k* is the number of significant edges and *N* is the number of parcels, *N =* 400). Similarly to the amplitudes, the connection densities were visualized in the TF plane. Graph theory (Bullmore and Sporns, 2009) was then used to characterize the networks formed by statistically significant parcel-parcel phase synchrony. Here, parcels constitute the nodes and significant synchronization the edges of the network.

### Visualization of the topography of amplitudes and network synchrony

To identify the brain with the most prominent effects in the time or TF window-of-interest (TFROI), we displayed the fraction of significant TF-elements of all elements for each anatomic parcel, visualized on a representative inflated cortical surface (*P^+^_TF_/P* ^−-^*_ΤF_*; Fig. 1*j*). Functional intrinsic network borders based on population level fMRI resting state activity (Yeo et al., 2011) were overlaid on the inflated surface as land marks.

Visualization of network synchrony is confounded due the linear signal mixing caused by inaccurate source reconstruction. We employed several new approaches to overcome this issue. We first assessed the reliability of source reconstructions and estimated interactions (Korhonen et al., 2014). To decrease the probability of reporting artificial and spurious synchronization, we removed from the analyses parcels with source reconstruction accuracy (fidelity) lower than 0.11. Next, we excluded parcels that were prone to include oculomotor artefacts in MEG. The removed parcels were mostly located in orbital frontal, anterior and inferior temporal and medial structures (Fig. 1*l*).

Linear signal mixing also introduces artificial and spurious correlations into pairwise metrics of sensor or reconstructed source MEG data (Palva and Palva, 2012). Although iPLV is insensitive to zero- and π-phase lag coupling, it is sensitive to spurious interactions, i.e., false positive connections arising from the signal mixing with neighbor of parcels with a true phase-lagged connection. Here, we used a novel edge-bundling approach to group edges into bundles by their functional adjacency in linear mixing space so that the goal for bundling is to hierarchically cluster connections into groups that collectively reflect the true connections (Siebenhühner et al., 2016). Such edge bundling results in a simplified and more appreciable graph with more reliable estimation of true edges and graph properties (Fig. 1*o*). For visualization, the resulting graphs (Figs. 5, 6) were colocalized with the seven functional brain systems of the Yeo parcellations (Yeo et al., 2011; Fig. 1*p*).

### Estimation of synchronization patterns across different distances

We computed normalized Euclidean distances for each pair of cortical parcels to assess the anatomic distance distribution of observed synchronization in the TF windows of interest. We selected the synchronization distances for each significant parcel pair from the average cortical distance-map based on the population mean of all the subjects in the study (*n* = 35). Distance map comprised all the Euclidean distances derived from the RAS-space for each possible combination of 400 parcels yielding in total 160,000 distance values. These values were normalized by the longest possible distance on the whole cortex and the normalized Euclidean distances were binned into five bins. We then estimated the distances for the significantly synchronized parcel-pairs and the proportion of synchronization in each bin. These data were compared against a surrogate distance distribution that was built by randomly taking 5000 times the same number of edges and its 95% confidence intervals (Fig. 1*s*).

### Correlation with clinical symptoms

To explore the putative links between interareal phase synchrony and clinical scores, we tested whether graph strength (GS) in the mid-γ (40–51 Hz) band response covaried with PANSS ratings and with the Z-score sum of the neuropsychological scores. To estimate individual *GS* values, we first computed individual weighted graphs by multiplying individual baseline corrected iPLV interaction matrices with a binary mask based on group graphs, *M(Group)*. Parcels and edges removed for low reconstruction accuracy or at-risk for oculomotor artifacts were excluded by masking (*M(DEM)*) from this analysis as well. Binary masks were defined for the mid-γ (40–51 Hz) band and 125- to 325-ms time window for the contrast of upright versus inverted Mooney stimuli and controls ScZ (Fig. 6*B*). If the interaction at frequency *f* and in time window *t* was found significant between parcels *p* and *q* in the group-level analysis, *M(p_,_ q, m, t, f)* was set to 1, otherwise to 0. For each subject, we multiplied adjacency matrices with these masks and then summed over all parcel pairs. Subjects’ individual *GS* for the time and frequency window of interest was thus calculated as:$GS=\sum_{i=t,f}^{N}{\left(M(Group\right)}_{\mathrm{i.}}*{M\left(DEM\right)}_{\mathrm{i.}}*S_{i})$, where M(x) are binary masks as defined above and S synchronization strength in the given time (*t*) and frequency window (*f*) for total of N windows and .* indicates the Hadamard (entrywise) matrix product. *GS* values were then sorted according to clinical scores and plotted as a function of increasing scores. Pearson correlation was used to estimate the correlation and bootstrapping with 10,000 surrogates to estimate confidence limits (Fig. 1*r*).

### Summary of statistical analyses

**Figure 3.**
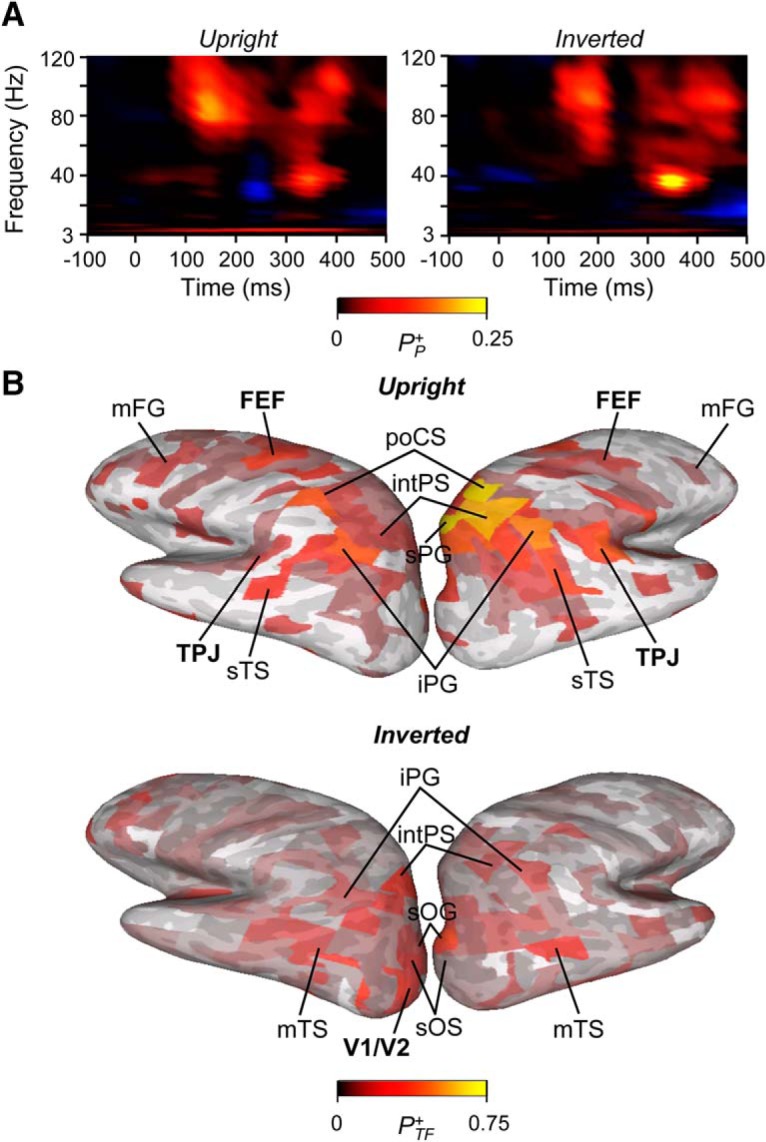
TFRs of significant oscillation amplitude modulations. ***A***, Difference in oscillation amplitudes between controls (CS) and ScZ patients for the correctly perceived upright and inverted stimuli (Welch’s *t* test, *p* < 0.05, corrected). Color scale indicates the fraction of brain regions with a significant positive CS-ScZ difference. The fraction of significant negative effects was negligible (blue colors). ***B***, Cortical regions in which significant differences in oscillation amplitudes between the CS and ScZ were observed for the selected TF region of interest indicated by rectangles in ***A*** for upright and inverted stimuli displayed on inflated cortical surfaces. Colors of the parcels indicate the fraction of TF elements with significant modulation in the parcel. Acronyms for the anatomic and function brain areas: FEF, frontal eye field; iPG, inferior parietal gyrus (here angular gyrus); mFS/G, middle frontal sulcus/gyrus; mTS, middle temporal sulcus; poCS, postcentral sulcus, sOS/G, superior occipital sulcus/gyrus; sPG, superior parietal gyrus; sTS, superior temporal sulcus; TPJ, temporoparietal junction; V1/V2, primary/secondary visual cortex.

**Figure 4.**
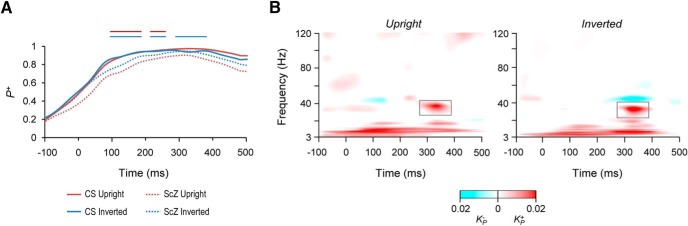
Interareal stimulus induced synchronization is stronger for the SC than for ScZ. ***A***, The extent of ERs across cortical parcels for the upright and inverted stimuli separately for the CS and ScZ subjects. The *y*-axis displays the proportion of cortical regions in which the ER was stronger than in baseline (Wilcoxon signed ranked test, *p* < 0.05, FDR corrected). The lines above indicate significant differences between the groups (Welch test, *p* < 0.05, FDR corrected). ***B***, The extent of significantly different interareal synchronization between SC and ScZ. TFRs show synchronization separately for upright and inverted stimuli as estimated with iPLV. Color scale indicates the connection density (*K*), e.g., the proportion of statistically significant connections of synchrony from all possible connections between groups (Welch’s *t* test, *p* < 0.05 FDR corrected).

**Figure 5.**
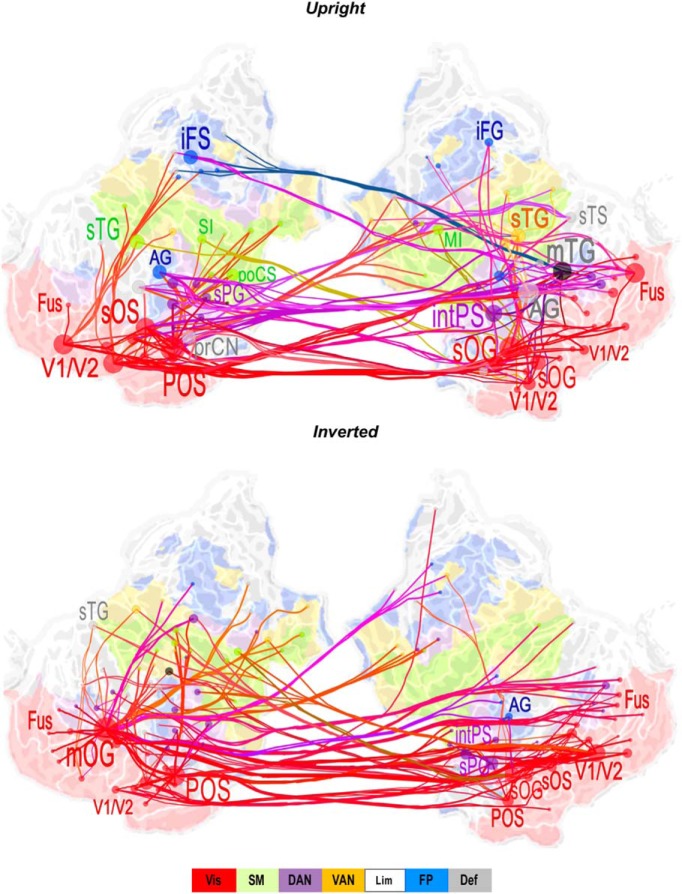
Cortical networks of low-γ-synchronization that differ between CS and ScZ. ***A***, Low-γ-band networks that were stronger for controls than ScZ subjects for the upright and inverted stimuli in the TF ROI of 30 − 40 Hz and 150 − 350 ms. Graphs display 200 strongest connections on an inflated and flattened cortical surface. Colors and abbreviations as in Figure 2. AG, angular gyrus; iFS/G, inferior frontal sulcus/gyrus; MI, primary motor cortex; mOG, middle occipital gyrus; mTG, middle temporal gyrus; Fus, fusiform gyrus; POS, parieto-occipital sulcus; prCN, precuneus; SI, primary somatosensory cortex; sTG, superior temporal gyrus.

**Figure 6.**
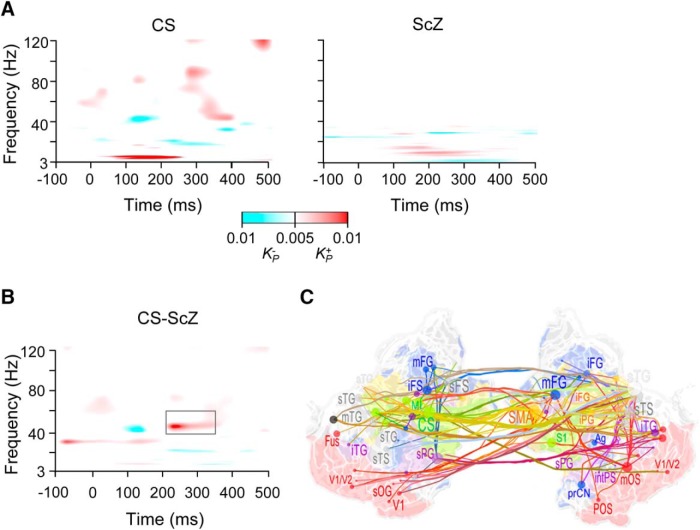
γ-Band synchronization reflects perceptual binding in CS and is stronger in CS than in ScZ. ***A***, TFR for the difference in the strength of parcel-to-parcel synchronization between correctly perceived upright and inverted trials separately for CS and ScZ as estimated with iPLV (Wilcoxon tests, *p* < 0.05, FDR corrected). ***B***, TFR for the difference between CS and ScZ groups in the upright-inverted contrast (Welch’s *t* test, *p* < 0.05 FDR corrected). Rectangle indicate the ROI selected for visualization in ***C***. ***C***, Mid-γ-band (40–50 Hz) network that for the upright-inverted contrast that was stronger for CS and ScZ group in the 200- to 300-ms time window (the graph is displayed as those in Fig. 5, abbreviations as in Figs. 3, 5). CS, central sulcus; iTG, inferior temporal gyrus; SMA, supplementary motor area.

All statistical analyses are described in detail in respective positions of the Materials and Methods section and summarized here. Primary statistical analyses between conditions were performed either with two-sample *t* test, or Welch *t* test of analysis of covariance for all time windows and frequencies. To correct for multiple comparisons, we discarded the number of least significant observations that was predicted by the α-level and visualized only the observations that were above this threshold (Figs. 3*A*, 4*A*, 6*B* ). The exact *p* values in the TFRs are not reported because of the large number of individual observations. To minimize the number of false positive connections (Figs. 5, 6C), we used several novel approaches. We first removed from the subsequent analyses the parcels with low source reconstruction accuracy or parcels that were prone to detect oculomotor artefacts. We then used a novel edge-bundling approach to group edges into bundles by their adjacency in the linear mixing space, which both inherently reduces the fraction of false positives and illustrates the most likely and statistically robust true neuronal connections.

## Results

### Behavioral performance

In the control group, HR for upright stimuli was 81.6 ± 3.7% and for Inverted stimuli 85.3 ± 6.7%. In chronic ScZ patients, HR for upright stimuli was 73.8 ± 4.4% and for Inverted stimuli 82.5 ± 6.4%. HR for upright stimuli was significantly lower for the ScZ than for the control group (*p* < 0.05, one-way ANOVA followed by *post hoc t* tests with Bonferroni correction; Fig 2*B*). For both ScZ and controls, RTs were shorter for upright (0.64 ± 0.04 and 0.59 ± 0.04 s, respectively, for ScZ and controls) than Inverted Mooney stimuli (0.77 ± 0.09 s and 0.83 ± 0.1 s; one-way ANOVA followed by *post hoc t* tests with Bonferroni correction; Fig 2*B*).

### Local γ-band oscillations are reduced in chronic ScZ

In our previous study, we observed reduced local γ-amplitudes in ScZ patients in the current task using sensor TF representations (TFRs) estimates (Grützner et al., 2013). To confirm these observations with a source-level analysis, we analyzed amplitude modulations across frequency bands separately for both healthy control and ScZ groups. These data were summarized as TFRs which show the fraction of brain areas, parcels, in which the modulation of oscillation amplitudes was statistically significant (Wilcoxon signed-rank test, *p* < 0.05 FDR corrected) compared to the prestimulus period. In line with previous observations at the sensor-level (Grützner et al., 2013), we observed an early amplitude modulation in both low-γ (30 − 51 Hz) and high-γ (60 − 120 Hz) bands, which was stronger for the controls than for the ScZ patients during the poststimulus period for both upright and inverted stimuli (Fig. 3*A*; Welch test, *p* < 0.05, corrected). We then identified the cortical sources that showed the strongest reduction of high γ-band oscillations in ScZ patients compared to the control group separately for upright and inverted conditions. We observed reduced γ-oscillations in ScZ patients for both stimulus conditions in occipital cortex in superior occipital gyrus and sulcus (sOG/S) as well as superior and middle occipital gyri and sulci (s/mOG and s/mOS) but also in intraparietal sulcus (intPS). For the upright condition, ScZ-related reduction in γ-oscillations was also in the temporoparietal junction (TPJ) and superior precentral sulci (sprCS, corresponding to the frontal eye fields, FEF) as well as in middle frontal gyrus (mFG) of the lateral prefrontal cortex (lPFC; Fig. 3*B*).

Interareal synchronization in the lower frequencies from delta to α-bands may be confounded by stimulus locked phase-synchronization due to the ERs (Palva and Palva, 2012). We therefore also computed the strength of ERs compared to baseline for both conditions and for both groups as well as the difference in the strength of ERs between the groups. These data were then visualized as the fraction of parcels where the modulation of oscillation amplitudes was statistically significant (Wilcoxon signed-rank test, *p* < 0.05, FDR corrected; Fig. 4*A*). For both upright and inverted conditions, ERs were indeed significantly stronger in controls than in ScZ patients.

### Interareal phase synchrony differs between controls and ScZ patients

Our main aim was to investigate whether large-scale synchronization would reveal dysconnectivity anomalies in ScZ patients. To this end, we quantified stimulus-induced interareal phase synchronization between all cortical parcels with the iPLV and to exclude the possible contribution of stimulus evoked activity and synchronization (Fig. 4*A*), compared these synchronization estimates against those obtained with forward-inverse-modeled surrogate data (see Materials and Methods). We used graph theoretical notation (Bullmore and Sporns, 2009; Rubinov and Sporns, 2010) to visualize interareal synchrony so that significant connections were represented as edges and parcels as nodes. As with the oscillation amplitude data, we summarized interareal synchronization data using TFRs to indicate the proportion of significant connections from all possible connections (connection density, *K*).

Controls had stronger phase-synchronization compared to ScZ patients in several frequency bands and at different epochs after stimulus onset for both upright and inverted stimuli: in the θ (4 − 8 Hz) and α (8 − 12 Hz) frequency bands between 0 and 400 ms from stimulus onset and in the low-β (14 − 20 Hz) and low-γ (30 − 40 Hz) at around 350 ms (Welch test, *p* < 0.05 FDR corrected; Fig. 4*B*). In addition, for the inverted stimuli we observed weaker synchronization for controls compared to ScZ patients in between 40 and 51 Hz. Importantly, these differences in large-scale synchronization remained significant after removing correlations present in the surrogate data and those exhibiting zero-phase-lag synchronization. However, synchronization in the θ-band was observed at the same latency window than visual ERs and amplitude modulation (Palva et al., 2011; Fig. 2*A*). Accordingly, θ-band synchronization likely reflects evoked activity rather than true induced phase-synchronization.

### Long-range γ-band synchronization connects the nodes in the visual system and frontoparietal network

To identify group differences in the anatomic layout of synchronization networks in the low-γ frequency (30 − 40 Hz) band, we identified the most central interareal connections and the key cortical areas, i.e., the network hubs (see Materials and Methods). For both the upright and inverted conditions, low-γ−band synchronization was increased for controls compared to ScZ patients between left and right-hemispheric visual cortices, specifically between visual areas V1/V2 and several nodes in the lateral occipital cortex (LOC) in both hemispheres including fusiform gyrus (Fus; Fig. 5). Importantly, V1/V2 and LOC were also strongly connected to inferior frontal sulcus/gyrus (iFS/G) of the lPFC and intPS of the posterior parietal cortex (PPC). These regions belong to dorsal (DAN) and frontoparietal (FPN) attention networks. Importantly, the fusiform gyrus was a hub in the γ-band networks only in the upright condition compared to inverted Mooney faces.

### γ-Band synchronization is correlated with perceptual organization only in controls but not in ScZ patients

As ScZ is associated with deficits in the integration of visual features into coherent object representations (Uhlhaas and Mishara, 2007), we also investigated the specific networks underlying perceptual organization through comparing differences in interareal synchronization between upright and inverted stimuli in the two groups. Controls showed increased mid-γ-band synchronization (40 − 51 Hz) for upright compared to inverted conditions between 300–400 ms, whereas this increase was absent in ScZ patients (Fig. 6*A*). This differential modulation of phase-synchronization was significantly different between control and ScZ groups (Welch’s *t* test, *p* < 0.05, corrected; Fig. 6*B*) and involved phase-synchronization patterns between early visual areas V1/V2 and ventral stream (mFG/iFG) of the lPFC; Fig. 6*C*). In addition, we observed transiently stronger synchronization for upright than inverted stimuli in the θ-band but this increase in the θ-band did not differ significantly between controls and ScZ patients (Fig. 6*A*).

### Long-range synchronization impairments in ScZ patients

**Figure 7.**
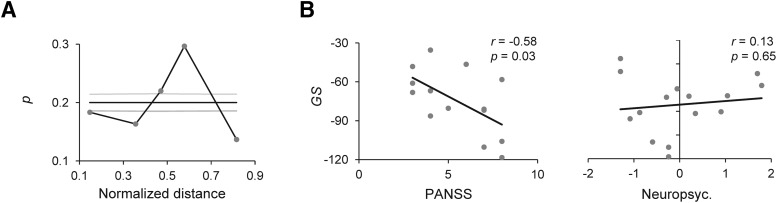
The severity of ScZ is correlated with the strength of mid-γ-band synchronization. Long-range γ-synchronization related to perceptual binding is reduced in ScZ. ***A***, The fractions of significant connections of perceptual (upright-inverted) mid-γ-band (40 − 51 Hz, 325 − 425 ms) synchronization (*y*-axis) divided into five connection-length bins according to their normalized Euclidian distance distributions (*x*-axis). The proportion of significant connections in each bin was estimated separately for the 300 of most significant connections and compared to parcel-shuffled surrogates. Black and gray lines indicate the mean and 2 SD of surrogate data. ***B***, The correlation of the GS with the disorganization PANSS and neuropsychological scores estimated for mid-γ-band GS (41 − 50 Hz, 325 − 425 ms) for the difference between upright and inverted stimuli.

We further asked whether the interareal mid-γ-band synchronization in the upright-inverted perceptual contrast, which was suppressed in ScZ patients, involved long- or short-range connectivity (Fig. 6). We estimated the parcel-parcel distances of 300 most significant connections and estimated the proportion of these connections in five equiprobable distance bins obtained with parcel-shuffled surrogate data. This analysis showed that the suppression of γ-band synchronization in ScZ patients was most pronounced over medium and long distances (3th and 4th bins; Fig. 7*A*).

### The strength of γ-synchronization is correlated with the severity of clinical symptoms of ScZ

In our previous study (Grützner et al., 2013), γ-band oscillation amplitudes were correlated with the severity of the PANSS “disorganization” factor. We thus established whether the changes in perceptual (upright-inverted) mid-γ-band (40–51 Hz) synchronization were also linked to clinical features of ScZ. We first estimated graph strength (GS) of the mid-γ-band networks in which synchronization was stronger for the upright than inverted trials in controls but not in ScZ patients (Fig. 6; Materials and Methods). We then estimated the correlation of individual *GS* values with clinical and neuropsychological scores. We found that *GS* in the 40 − 51 Hz range was negatively correlated with PANSS (*r* = −0.58, *p* < 0.03) but not with neuropsychological scores (*r* = 0.13, *p* = 0.65) (Fig. 7*B*). The correlation of *GS* with PANSS scores was significant and robust also when estimated with a randomization test (95% range for shuffled data: *r_shuffled 2.5%_* = −0.52 and *r_shuffled 97.5%_* = 0.52) and with bootstrapped confidence limits of the correlation coefficient per se (95% confidence limits *r_bootstrap 2.5%_* = −0.19 and *r_bootstrap 97.5%_* = −0.78), respectively.

## Discussion

Impaired cognitive and perceptual functions are a core aspect of ScZ (Green, 1996) but the neuronal mechanisms underlying these deficits are still unclear. One candidate mechanisms is an impairment in the synchronization of oscillatory activity between brain regions (Uhlhaas and Singer, 2012). This perspective is consistent with both current and historical perspectives that have highlighted a dysconnection syndrome, a failure in the functional integration of distributed neuronal activity, as a fundamental aspect of the disorder (Friston and Frith, 1995; Stephan et al., 2009).

In the present study, we applied advanced MEG methods for the analyses of local and interareal neuronal synchronization as well as graph theoretical measures for the assessment of the large-scale network structures to address this question. The results show that both local synchronization, as reflected in amplitude modulations, and large-scale γ-band synchronization are reduced in ScZ patients during a cognitive task requiring perceptual integration. This is in agreement with accumulating evidence that the synchronization of high-frequency oscillations is closely related to perceptual processes and higher cognitive functions during normal brain functions (Kim et al., 2016; Michalareas et al., 2016), the disturbance of which could lead to cognitive and perceptual deficits in ScZ. This is consistent with a large body of evidence that has emerged over recent years that changes in excitatory and inhibitory transmission, in particular deficits in parvalbumin-expressing (PV+) interneurons and NMDA receptors, constitute a key aspect of cellular abnormalities in ScZ that could give rise to impaired high-frequency oscillations and their synchronization (Kantrowitz and Javitt, 2010; Gonzalez-Burgos and Lewis, 2012; Lewis et al., 2012).

The present study provides critical evidence for a dysfunction in large-scale synchronization in ScZ through the combination of advanced methods of source localization and time series analysis of MEG-data that allow novel insights into the anatomic layout of phase-synchronization abnormalities in ScZ. Specifically, our findings show that abnormal long-range synchronization may constitute a core systems-level mechanism for the cognitive and perceptual deficits in ScZ. In line with earlier studies on visual perception (Spencer et al., 2008; Grützner et al., 2013), we observed reduced γ-band amplitudes in ScZ patients in temporal cortex, PPC, and lPFC for the upright condition and also in early visual cortices for inverted Mooney faces. This suggests that reductions in high-frequency activity in ScZ patients are mainly caused by deficits at later stages of the visual hierarchy, which would be in agreement with the evidence that perceptual closure involves higher visual areas (Grützner et al., 2010) that exert top-down control of visual information processing (Corbetta and Shulman, 2002; Corbetta and Shulman, 2011; Buschman and Kastner, 2015; Womelsdorf and Everling, 2015).

Previous studies have already provided preliminary evidence that long-range synchronization of rhythmic activity could be impaired in ScZ (Spencer et al., 2003; Uhlhaas et al., 2006). However, for the reasons summarized above, these findings are to be interpreted with caution because of the challenges in excluding the confounding factors such as volume conduction, non-neuronal artifacts, and lack of source identification (Schoffelen and Gross, 2009; Palva and Palva, 2012). The present study revealed also a reduction of phase synchronization for low-frequency oscillations (θ, α) in ScZ patients. As the occurrence of θ-band synchronization overlapped with amplitude and phase-modulation of evoked activity, it is conceivable that these deficits in ScZ patients may not reflect impairments in genuine large-scale synchronization (Palva and Palva, 2012).

In contrast, synchronization at high γ-frequencies and their reduction in ScZ patients were transient and reflect true induced phase-synchronization patterns which is supported by the analysis of surrogate date. Thus, it appears that both the temporal parsing of evoked responses and the long-range synchronization of these responses are impaired in ScZ patients. Whether the two alterations have a common cause or result from disturbances of different mechanisms is unclear. Support for the specific role of large-scale synchronization at γ-band frequencies in perceptual organization comes from the comparison of responses to upright versus inverted Mooney faces. Confirming previous data that suggested a specific role of γ-band oscillations in the construction of coherent object representations (Singer, 1999; Tallon-Baudry and Bertrand, 1999; Morgan et al., 2011; Grützner et al., 2013; Honkanen et al., 2015), we observed that controls exhibited a significant, transient increase in phase synchronization in the mid-γ-band range (40 − 50 Hz) at 300 − 400 ms, which was strongly reduced in ScZ patients. This reduction comprised interactions both within the visual system, e.g., among early visual regions and fusiform gyrus that underlie face perception (Haxby et al., 2002). Reduction was also observed between the visual system and key areas of the FPN and DAN in the PPC and lPFC that are involved in the coordination of visual attention (Corbetta and Shulman, 2002; Fox et al., 2005).

Moreover, our analyses revealed that reductions in γ-band synchronization in ScZ patients involved preferentially medium- and long-distance connections, providing support for the notion that the disorder is associated with impairments in the temporal coordination of distributed neural activity at global scales. Moreover, impairments of temporal coordination were correlated with the severity of the clinical symptoms, supporting the potential relevance of coordination failures in the emergence of clinical symptoms. Taken together, the present study, although not providing causal evidence, yields robust correlative support for the hypothesis that clinical symptoms and cognitive impairments in ScZ are associated with a dysconnection syndrome (Friston, 1998; Uhlhaas and Singer, 2012; Uhlhaas and Singer, 2015; Voytek and Knight, 2015).

### Future directions and limitations of the study

We observed reduced γ-band synchronization in chronic ScZ patients that were under antipsychotic medication. This could potentially constitute a confound for alterations in large-scale synchronization in the disorder. However, we have previously shown that reductions in high-frequency oscillations are present also in unmedicated, first-episode ScZ patients (Sun et al., 2013) suggesting that antipsychotic medication is not related to alterations in high-frequency activity. Furthermore, preliminary evidence suggests that alterations may be present before illness-onset in at-risk individuals (Tada et al., 2016). Future studies are required to determine if abnormalities in large-scale phase-synchronization predate the onset of frank psychosis and, as a result, could serve as a biomarker for early detection and diagnosis.

Additionally, the number of ScZ patients that entered the analysis is relatively small and replication in larger ScZ-samples is required. The differences observed in phase synchronization between groups, however, were obtained using a conservative data-driven statistical analysis approach and statistical observations were corrected for multiple comparisons. Accordingly, we are confident that the patterns of aberrant synchronization in the current dataset are robust indexes of dysfunctional large-scale networks in ScZ.

## Synthesis

Reviewing Editor: Bradley Postle, University of Wisconsin Decisions are customarily a result of the Reviewing Editor and the peer reviewers coming together and discussing their recommendations until a consensus is reached. When revisions are invited, a fact-based synthesis statement explaining their decision and outlining what is needed to prepare a revision will be listed below. Note: If this manuscript was transferred from JNeurosci and a decision was made to accept the manuscript without peer review, a brief statement to this effect will instead be what is listed below.

I have looked over your rebuttal letter, and it is my assessment that you have satisfactorily addressed all the concern raised by the reviewers who previously reviewed this manuscript. Therefore, it's my judgment that your manuscript does not require further peer review before being accepted for publication in eNeuro.
